# Current trends and future perspectives of stroke management through integrating health care team and nanodrug delivery strategy

**DOI:** 10.3389/fncel.2023.1266660

**Published:** 2023-11-15

**Authors:** Xuelu Han, Yingxin Qin, Chunli Mei, Feitong Jiao, Sanaz Khademolqorani, Seyedeh Nooshin Banitaba

**Affiliations:** ^1^Nursing Clinic, Affiliated Hospital of Jilin Medical University, Jilin, China; ^2^Department of Nursing, Affiliated Hospital of Jilin Medical University, Jilin, China; ^3^Nursing College, Beihua University, Jilin, China; ^4^Nursing Training Center, School of Nursing, Jilin Medical University, Jilin, China; ^5^Department of Textile Engineering, Isfahan University of Technology, Isfahan, Iran; ^6^Emerald Experts Laboratory, Isfahan Science and Technology Town, Isfahan, Iran; ^7^Department of Textile Engineering, Amirkabir University of Technology, Tehran, Iran

**Keywords:** stroke, healthcare team, professional nursing team, nanodrugs, nano carrier, blood brain barrier

## Abstract

Stroke is accounted as the second-most mortality and adult disability factor in worldwide, while causes the bleeding promptly and lifetime consequences. The employed functional recovery after stroke is highly variable, allowing to deliver proper interventions to the right stroke patient at a specific time. Accordingly, the multidisciplinary nursing team, and the administrated drugs are major key-building-blocks to enhance stroke treatment efficiency. Regarding the healthcare team, adequate continuum of care have been declared as an integral part of the treatment process from the pre-hospital, in-hospital, to acute post-discharge phases. As a curative perspective, drugs administration is also vital in surviving at the early step and reducing the probability of disabilities in later. In this regard, nanotechnology-based medicinal strategy is exorbitantly burgeoning. In this review, we have highlighted the effectiveness of current clinical care considered by nursing teams to treat stroke. Also, the advancement of drugs through synthesis of miniaturized nanodrug formations relating stroke treatment is remarked. Finally, the remained challenges toward standardizing the healthcare team and minimizing the nanodrugs downsides are discussed. The findings ensure that future works on normalizing the healthcare nursing teams integrated with artificial intelligence technology, as well as advancing the operative nanodrugs can provide value-based stroke cares.

## Graphical abstract

**GRAPHICAL ABSTRACT:**
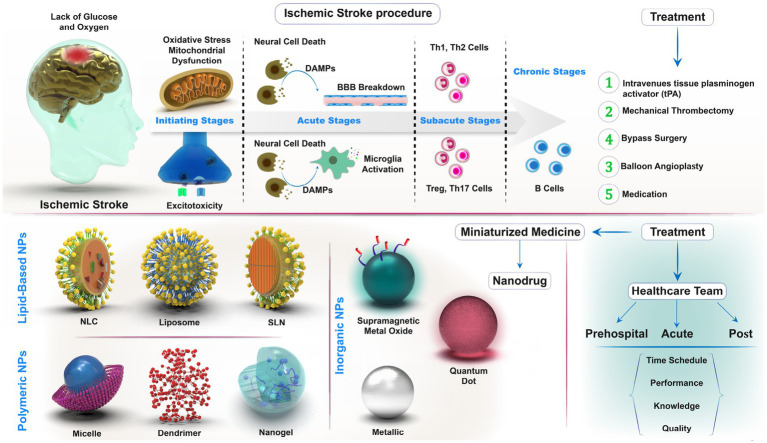

## 1. Introduction

**Figure 1:**
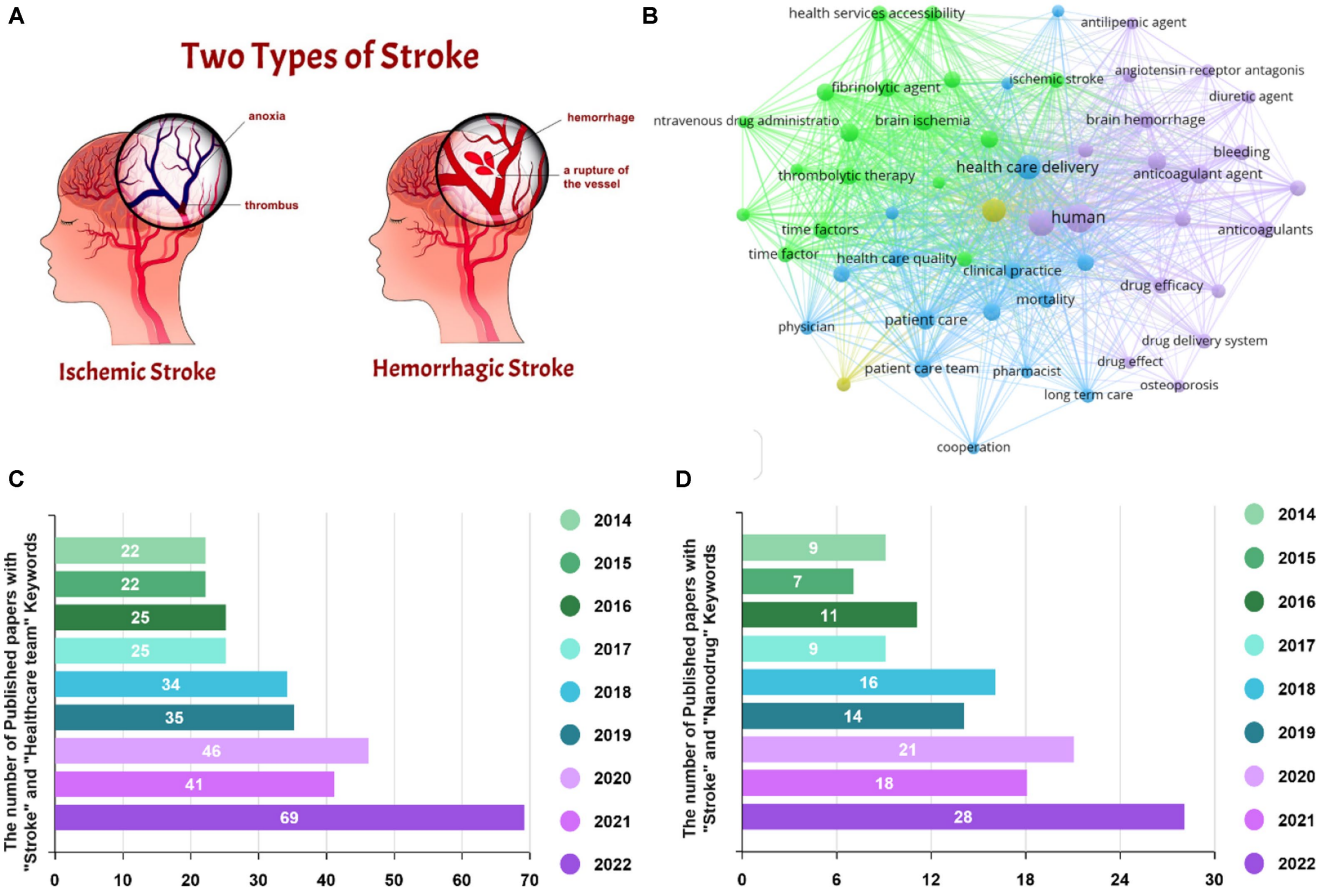
**(A)** The stages of stroke pathophysiology. Reproduced from Correa-Paz et al. (2021) with MDPI Copyright, **(B)** VOSviewer map for stroke, and the number of Scopus-indexed publications regarding the role of **(C)** healthcare team and **(D)** nanodrug delivery in stroke treatment.

Stroke is known as a medical condition that causes both physical and psychological disorders. According to World Health Organization, it affects about 15 million individuals annually, leading to a major mortality rate worldwide (Chemerinski and Robinson, 2000; Wolfe, 2000). Stork could be classified into two main categories of hemorrhage and ischemia, formed by blood vessels’ bleeding and clotting, respectively (see Figure 1A; Correa-Paz et al., 2021). Hemorrhagic stroke is responsible for 10–20% of all strokes, contributes to 40% of stroke-related mortalities, and reasons a major rate of disabilities, receiving an increasing concern. The cerebral parenchyma and bleeding in the sub-arachnoid cavity are two kinds of hemorrhage, recognized as intracerebral hemorrhage (ICH) and subarachnoid hemorrhage (SAH) (Lee et al., 2022).

Based on the literature, few impactful therapies have been declared for bleeding stroke, leading to poor clinical outcomes. Meanwhile, FDA has approved several strategies, including intravenous administration of recombinant tissue plasminogen activators, endovascular thrombectomy, thrombolytic agents, and mechanical intervention, which cause clot recovery and repair fusion. It should be noted that the aforementioned ways are time-consuming or require surgical equipment, while prompt and coordinated care is mandatory to optimize patient consequences. Considering the challenges, the healthcare team plays a critical role in the early management of stroke patients and ensuring that they receive appropriate follow-up supportive care. Recent attempts have also declared the promising role of miniaturized drugs toward crossing the blood brain barrier (BBB) integrity and providing a proper thrombolytic agent (Candelario-Jalil et al., 2022; Nilles et al., 2022).

The healthcare team typically includes emergency nursing services, emergency department physicians and nurses, neurologists, radiologists, rehabilitation specialists, and social workers, representing crucial impacts in various clinical stages. The emergency nursing team recognizes and triages the patient in the first step, causing an influential parameter in the outcome. The construction of a specialized stroke healthcare team can realistically assist stroke emergency nurses to gain the theoretical knowledge and clinical expertise of stroke resuscitation work, enhance the features and capabilities of the stroke resuscitation procedure coordination, and eventually enable a highly efficient stroke emergency (Koç et al., 2022; Verma et al., 2022). By emerging nanotechnology, innovative drug delivery systems have been employed to cure various diseases through boosting delivery efficiency, as well as minimizing side effects (Fang et al., 2015, 2016). Nanodrug systems allow for the improvement of pharmacological and pharmacokinetic paradigms of traditional medicines or targeting drug deliveries by operating as pharmaceutical carriers (Wang et al., 2010; Fan et al., 2019). New advancements in nano-pharmaceuticals to induce hemostasis, nerve protection, comorbidity prophylaxis, and neurological revitalization suggest pioneering ideas for managing hemorrhagic strokes (Li et al., 2021; Tian et al., 2021). Visualizing map for stroke is depicted in Figure 1B, corroborating the impact of both the healthcare team and drug delivery systems to survive stroke patients. The number of Scopus-indexed papers considering the effect of the healthcare teams, as well as the drug delivery systems is also attested in Figures 1C,D, illustrating the increasing focus of the research endeavors. Therefore, in this review paper, we first overviewed the main impact of the healthcare team on caring the stroke patients. With the aim of boosting outcomes of curative approach, the effectiveness of the medicine has been recommended by drug downsizing. So, the performance of nanodrugs toward obtaining appropriate results is highlighted. Finally, the challenges and future directions are provided.

## 2. The importance of the healthcare team in treating stroke cases

Stroke causes a serious long-term disability, which may affect emotional, physical, communal, and financial consequences involving patients, along with their family members and friends. Take the advantage of a well-organized stroke care unit and a professional nursing team could lead to a notable treatment for stroke patients. Nursing is a specialization in the healthcare field to reach sustain and optimal health and well-being systems, encompassing all individuals and communities. The nurse care model originated in the United States and is a theory form that could result in a persuasive guidance for nursing practices. Nurses have shown an integral role in performing the management of stroke patients. With the development of stroke therapy teams, stroke patient care groups are led by trained nurses, who serve an essential function in enhancing stroke patient prognosis (Burns et al., 2022; Verma et al., 2022; Arulogun et al., 2023).

**Figure 2:**
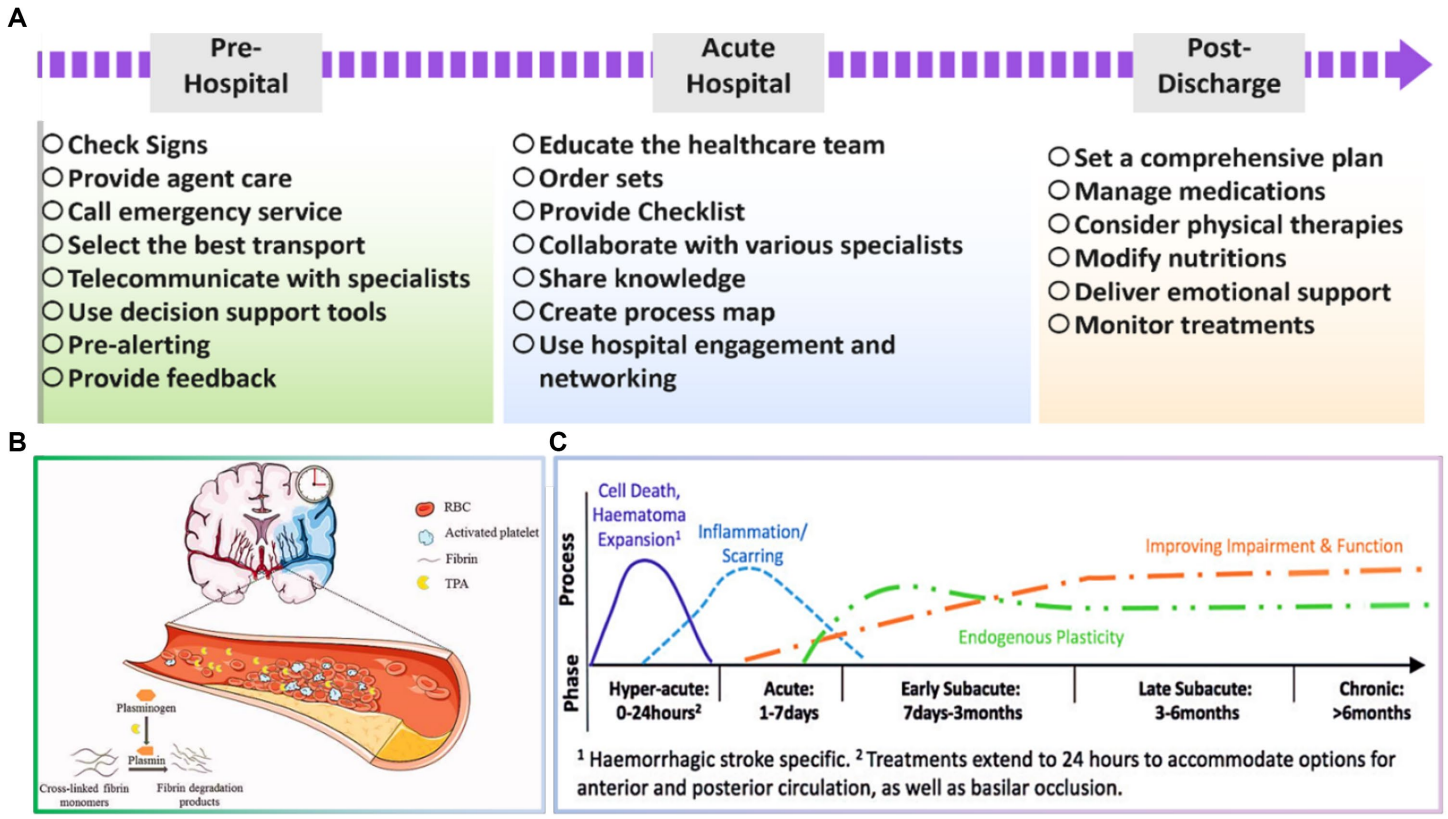
**(A)** Essential tasks in various stages toward successful stroke treating, **(B)** schematic illustration of acute ischemic stroke. Reproduced from Ma et al. (2021) with Taylor & Francis Copyright, and **(C)** timing of vital processes during the first 6 months after stroke. Reproduced from Bernhardt et al. (2017) with SAGE Copyright.

According to the clinical demands, the stroke healthcare teams will engage and coordinate the therapeutic processes of patients. The professional stroke care team is a vital part of stroke emergency medical staff team, which is very valuable in increasing the thrombolysis rate and decreasing the disability rate and stroke mortality. The UK, USA, and other developed countries have settled a wide range of advanced practitioner nurse groups regarding stroke, such as specialized emergency and specialized rehabilitation nurses, greatly influencing the management and rehabilitation of stroke sufferers (Cormican et al., 2023; Maciel Barbosa et al., 2023). Accordingly, stroke as a complicated clinical condition obligates health experts to benefit from their specialist knowledge and skills for the stroke survivors. Nevertheless, stroke centers in China have been in the initial stage of growth, in which the lacking of human resources is sensed (Dubach and Tripathi, 2023; Nogles and Galuska, 2023). Additionally, stroke specialized healthcare team is a novel concept, so its operation has no mature governance system. Thus, competence-based medical education and personnel training have increasingly become the key focus toward growing the healthcare human resources. The number of stroke patients discharged from hospitals and the per capita expenditure on healthcare has increased steeply from 2005 to 2017, as reported in the 2018 China Health Statistics Report (McGlennen et al., 2023). Relevant expert studies have presented that the gross cost of a single stroke sufferer consultation, medication, and rehabilitation is approximately 50,000 yuan per year (Lossius and Lund, 2012). Therefore, professional treatment by the healthcare team is essential in the early pre-hospital stage, as well as acute hospital and post discharge steps to incline the success rate of stroke treatment and reduce the mortality and disability. Figure 2A summarizes significant cares which should be assumed in each stage. More information regarding the important factors in each process is highlighted in the following.

The Materials and Methods should be described with sufficient details to allow others to replicate and build on the published results. Please note that the publication of your manuscript implicates that you must make all materials, data, computer code, and protocols associated with the publication available to readers. Please disclose at the submission stage any restrictions on the availability of materials or information. New methods and protocols should be described in detail while well-established methods can be briefly described and appropriately cited.

Research manuscripts reporting large datasets that are deposited in a publicly available database should specify where the data have been deposited and provide the relevant accession numbers. If the accession numbers have not yet been obtained at the time of submission, please state that they will be provided during review. They must be provided prior to publication.

Interventionary studies involving animals or humans, and other studies that require ethical approval, must list the authority that provided approval and the corresponding ethical approval code.

### 2.1. The intervention necessity for stroke patients in the early pre-hospital stage

The pre-hospital stage of the stroke is a critical period in stroke management, as it is the time between the onset of stroke symptoms and the arrival of the patient at the hospital. During this stage, it is vital to recognize the signs of stroke, provide agent-care in place, and call emergency services immediately. Additionally, the strategies used for the pre-hospital transport, networked stroke care, and real-time feedback systems are vital to be considered by the healthcare team (Lossius and Lund, 2012; Kobayashi et al., 2018). The early recognition could be attained through various symptoms, including sudden weakness or numbness in the face, arm, or leg on one body side, immediate confusion, trouble speaking, difficult speech, trouble seeing in one or both eyes, dizziness, loss of balance or coordination, severe headache with no known cause (Kamal et al., 2022; Ospel et al., 2022).

Acute ischemic stroke and the importance of time-dependent tissue plasminogen activator (tPA) treatment are schematically shown in Figure 2B. There is a strong relation between time from onset of treatments and irreversible tissue damage. So, selecting the most appropriate strategy for arriving at the patient and providing early services could be significant. In this period, the early recanalization of an occluded vessel is a very effective means of salvaging tissue at risk for stroke to preserve brain tissue until the blood supply is reestablished. Moreover, the transport modality to the emergency department is also influential for the stroke patient’s recognition, triage, and treatment. It is widely reported that the task should be carried out within the golden time, which is around 60–90 min. Therefore, an expert team should promptly select the best transport method, ground or air medical emergencies, regarding their situation (Setianingsih et al., 2019; Lumley et al., 2020; Damon et al., 2022). Sveikata et al. (2022) declared that the positive predication value was inclined from 54.8 to 79.8% through employing interactive emergency medical services training, which caused a significant reduction in mortality rate of stroke cases, highlighting the importance of training in pre-hospital stage. In a case study carried out in Taiwan, Hsieh et al. (2016) presented that a close cooperation between the emergency medical service and the hospital could result in a sooner thrombolytic therapy and therefore and an improved in-hospital stroke care. Meretoja et al. (2017) have also demonstrated that an additional week of patient health is obtained for endovascular interventions as the delay time is decreased by 1 min. Consequently, it is critical to enable blood flow reconstruction as speedily as possible to improve the efficiency of in-hospital therapy.

Overall, a well-arranged healthcare team in this step could inhibit recurrent stroke, optimize life quality, and diminish late complications. In this era, the performance could be boosted through several recommendations, including hospital pre-alerting, implementing telemedicine with professional medical teams, fast data collection and online transferring to the hospital, embedding modern diagnostics in ambulances, and so forth. Regarding this, Audebert et al. (2013) remarked that equipping ambulances with telemedicine, CT scanners, and point-of-care laboratories, as well as the presence of dispatchers and paramedics could open new perspectives toward recognizing and therapy of patients before arriving at the hospital. This period prepares a proper condition to obtain successful outcomes in acute hospital care.

### 2.2. The role of the specialized stroke care team in hospital

Hospital care is decisive for treating stroke as it provides access to specialized medical professionals, equipment, and facilities necessary for timely and operative stroke management. Stroke patients must receive prompt and appropriate care to minimize brain damage, prevent complications, and improve their chances of recovery. In the hospital setting, stroke patients may undergo a range of diagnostic tests, such as computed tomography (CT) scans or magnetic resonance imaging (MRI), to determine the type and location of the stroke. Based on the results obtained from these assays, the healthcare team can develop an individualized treatment plan that may include medications to dissolve blood clots or prevent further clot formation, blood pressure and cholesterol management, and rehabilitation services such as physical, occupational, and speech therapy (Webb et al., 1995; Audebert et al., 2017). In some cases, stroke patients may require more advanced interventions, like endovascular thrombectomy, which involves removing a blood clot from the brain using a catheter, or neurosurgery to repair damaged blood vessels or eradicate a clog. Hospital care also provides the opportunity for stroke patients to receive ongoing monitoring and support, including regular neurological assessments, medication adjustments, and education on stroke prevention and management (Bershad et al., 2008; Audebert et al., 2009).

Stroke specialty nursing team informatics facilitate the completion of the regulatory process for stroke emergency specialty nurses. Recent domestic and international studies have shown that the use of time tracking sheets for quality control and regular retrospective summary meetings are beneficial to optimize the process. Domestic and international scholars have proposed the concept of time goal management (Audebert et al., 2006; Abdul Aziz et al., 2017). As an example, a hospital was adopted a stroke timer strategy to greatly reduce the time from admission to CT and door to needle time (DNT). The transactive surveys and multivariate regression findings have shown that the delay in critical parts of the in-hospital stroke management is due to timely deployment of the stroke group, inadequate convergence of the multiple parts, and deferred decision-making on the affected side. With the direction of the stroke specialized care team, the stroke emergency specialized nurses require advanced professional knowledge, in-advanced assessing, and quick communication with specialists to enable the physicians to concentrate on therapeutic decisions (Liu et al., 2018). Gustavo et al. (2005) declared that a complete recovery could be obtained after 3 months in the case of obtaining advantageous data, monitoring the glucose level, attaining real-time cortical CT scan, and checking the response of the neurological system in the first 24 h after stroke. Additionally, proper management on the administration of tissue plasminogen activator and appropriate discharge planning were advised. Nor et al. (2005) also figured out that the emergency room has two main roles in acute hospital stroke treatment. First, as time is really important, fast recognition and triage could enhance thrombolysis success. Then, a significant reduction could be observed in the non-stroke referral to a professional stroke unite when receiving valid data in a well-equipped emergency room.

In a case study carried out in 2014 for 145 base hospitals in China, a multidisciplinary joint prevention and treatment model was established. In this model, the stroke emergency nurse specialist provides dynamic feedback on the process and promotes continuous optimization of stroke hospital care during the construction of the stroke center, which is of far-reaching significance for the sustainable and benign development of stroke centers. Accordingly, the allocation of nursing resources and the level of care in stroke centers varies from region to region (Damon et al., 2022). Also, the specialist stroke nurse position does not allow the nurses to play a full role in making multidisciplinary team connections, assisting in treatment decisions, and monitoring the efficiency of team members. Therefore, although the model is replicable, there is a crucial need for further development of specialized nurse human resources and construction to provide a versatile model (Sveikata et al., 2022). While the overall efficiency of in-hospital treatment of most strokes in China has reached the standard, there is still a gap compared to the advanced international level (Bershad et al., 2008). Strategies such as multi-consultation prediction education shorten medical decision-making time, which in turn guides the efficient operation of the hospital stroke treatment process (Audebert et al., 2009). In addition, the managerial role of stroke emergency nurses should be ensured in terms of core competency training, scheduling plan adjustment, construction of mobile nursing teams for stroke care, improvement of information technology and system construction, and optimization of hardware facilities.

According to the literature, the hospital care stage could be enhanced in several ways, including telestroke services, artificial intelligence, wearable devices, prevision medicine, and virtually reality. Telestroke services are known as emerging approaches toward improving stroke care in hospital setting, allowing remote access to neurological expertise and specialized care through video conferencing and other communication technologies. This advancement could be really helpful in appropriate stroke care, particularly in rural or underserved areas. Artificial intelligence also has the potential to boost stroke treatment by providing real-time decision support to healthcare servers. Benefiting from artificial intelligence, the obtained data from patients could be analyzed to attest insights on the most effective treatment strategies, potentially improving patient outcomes, and reducing healthcare costs. Moreover, smartwatches and fitness trackers can continuously monitor vital signs and other physiological parameters, allowing for early detection and intervention in case of a stroke. Additionally, stroke care can be personalized to individual patients, potentially improving treatment outcomes and reducing the risk of complications through using precision medicine approaches. Furthermore, virtual reality technology is able to create immersive environments that simulate real-life scenarios, offering stroke patients with opportunities for rehabilitation and cognitive training (Audebert et al., 2006; Abdul Aziz et al., 2017; Ryan et al., 2017).

### 2.3. The role of healthcare team in post-discharge phase

Stroke is a debilitating condition that can long-term effect on a patient’s physical, cognitive, and emotional well-being. Therefore, it’s crucial to have a coordinated and comprehensive plan for post-discharge care to ensure that stroke patients receive the necessary support and resources to recover and manage their condition (Phillips et al., 1999; Deen et al., 2016). The healthcare team typically includes physicians, nurses, physical therapists, occupational therapists, speech therapists, social workers, and case managers, working together to offer a multidisciplinary approach and address the various needs of stroke patients (Yam et al., 2012; Gaire, 2018). Some of the specific roles of the healthcare team in post-discharge care for stroke patients include medication management, rehabilitation, dietary modifications, emotional support, and care coordination (Talbot et al., 2023; Yen et al., 2023). Figure 2C represents the timing of the significant tasks which should be considered across 6 months after stroke (Bernhardt et al., 2017). In other words, the healthcare team ensures that patients take their medications as prescribed and monitors for adverse effects or interactions. In addition, physical therapists, occupational therapists, and speech therapists work with patients to assist them to regain their physical and cognitive abilities (Ullah et al., 2020). Based on a study by Nguyen et al. (2021), physical activity and diet quality could remarkably improve the side effects of comorbidity on stroke patients with disability. Nutritionists or dietitians may also guide dietary modifications to prevent future strokes and manage underlying medical conditions, such as diabetes or high blood pressure. For example, Kishimoto et al. (2022) figured out that maintaining or gaining weight could be helpful during this stage in patients with a BMI lower than 30. Therefore, nutritional modifications have been extensively advised to inhibit immediate weight loss and observe better functional recovery. On the other hand, social workers and case managers may deliver emotional support to patients and their families and connect them with community resources, like support groups or counseling services. Eventually, the healthcare team works together to guarantee that there is a coordinated plan for post-discharge care and that patients have access to the necessary resources and support.

Considering the role of ideal healthcare team for stroke treatment, numerous advances have been employed in hospitals, specifically in developed countries. For example, Shanghai Changhai Hospital established the Cerebrovascular Disease Center in September 2013 by integrating the advantages of neurology and surgery. In addition, a cerebrovascular disease emergency, a stroke, and a treatment quality improvement team have been set to optimize the hospital care process continuously. The DNT implementation was also facilitated by anti-infection treatment in the emergency department and the establishment of a green channel for stroke treatment accompanied by specialists. Three emergency stroke nurses were trained at Shanghai Changhai Hospital to undertake direct anti-infection work for nursing patients. Although the standard has been met regarding stroke hospital treatment efficiency, there is still a gap compared to the international top level (Feng et al., 2017; Zhao et al., 2021; Yang et al., 2022). It is expected that with the emergence of nanotechnology, drug delivery systems based on nano-scales will affect the improvement of the treatment process and strengthen the results of the care and nursing department and the sets of activities taken for patient recovery. In other words, by increasing the effectiveness of the drug and its precise delivery, nurses can get much better results from their set of activities in stroke treatment. Therefore, in the next section, we will discuss the impact of nanotechnology on medicine and its targeted delivery systems.

## 3. Nanomedicines for stroke

Currently, routine therapies to manage stroke and correlated complications largely concentrate on drugs to remove the thrombus, as well as to minimize deleterious ischemic injuries to neurons via intravascular therapy or intravenous thrombolysis. Intravenous administration of rtPA in terms of thrombolysis was assumed to be the only commercially-available treatment until recently. At the same time, it was effective in a limited time and suffered the risk of intracerebral hemorrhages (Toni et al., 2008; Mehrpour et al., 2019). Recently, some newly developed thrombolytic agents, including the fibrin-particular tissue plasminogen activator, as well as the chimeric plasminogen activator, have been developed (Lv et al., 2022). Also, antiplatelet therapy and anticoagulants have been prescribed for secondary stroke prevention. In contrast, most antiplatelet drugs are capable of repairing or shielding the affected region from more injuries in no circumstances (Horlocker et al., 2003; Lv et al., 2022). According to the literature, the combination of antiplatelet and anticoagulants drugs with neuroprotective agents has been broadly suggested as a promising curative approach. Despite endowing several pros, the low permeability of neuroprotectants across BBB may lead to the failures of clinical transformation (Chamorro et al., 2016; Gaire, 2018). So, approaches to increase neuroprotectant accumulation in ischemia sites will not only ameliorate the curative efficiency but also contribute to their clinical translation.

Besides bypassing the physiological barriers, the site-specific drug release and co-delivery to ischemic areas as well as their decreasing side effects on other parts are major challenges of stroke treatments. For the next generation stroke therapeutics, it is hoped that downsizing the drug carrier through applying nanotechnologies, it will provide targeted and on-site delivery while effectively crossing through the BBB. Nanoscience, which emerged in 1959, is a new study branch relating to the formation of materials in the nanoscale range. This innovative technology has opened a new window in the recognition and therapy of various diseases. The nano-scaled materials have attested prompt and accurate diagnosis, as well as precise treatments with a significant reduction in the therapeutic doses and side effects. Additionally, it enables delivery of the drugs in targeted and desired regions. The application of nanomaterials in recognizing and therapy of the stroke cases is provided in this section.

### 3.1. Nanomaterials for stroke diagnosis

CT and MRI are well-known as beneficial tools for detecting the early phase of stroke, through providing structural imaging. With the growth and progress of technology, these methods are pursued to attain accurate thrombolysis and recanalization diagnosis in the brain after stroke. Rapid detection could benefit patients who require immediate treatment. Miniaturizing the employed contrast agents (Iron oxides) in MRI devices has prevented the passive leakage of particles and enhanced the material payload. Conjugating the MRI imaging with nanoscaled materials has clarified the capability of monitoring non-invasive post-stroke inflammation aspects. Enabling the control and monitoring of the inflammatory reaction allows the identification of the patient subsets, resulting in immunomodulatory therapies (Debatisse et al., 2020; Tian et al., 2021; Alonso-Alonso et al., 2022). As an example, Sillerud et al. (2020) applied novel iron-platinum nanoparticles conjugated with MRI, showing dynamic development monitoring of neuroinflammation during stroke progression in a living rat brain.

Although CT provides less sensitivity than MRI, its combination with nanomaterials could be employed as molecular imaging tool, through assessing the thrombus burden, as well as monitoring of thrombolytic therapy (Wang et al., 2015; Kim et al., 2016). In a study, Wang et al. (2015) illustrated that combining polyethylene glycolated BaHoF5 nanoparticles with CT could effectively enhance the accuracy and sensitivity of stroke recognition. Compared with iodinated contrast agents, which are the commonly applied agents in CT, these novel nanoparticles could decline the essential dose and provide imaging with higher efficiency.

### 3.2. The role of nanomaterials in treating stroke

**Figure 3:**
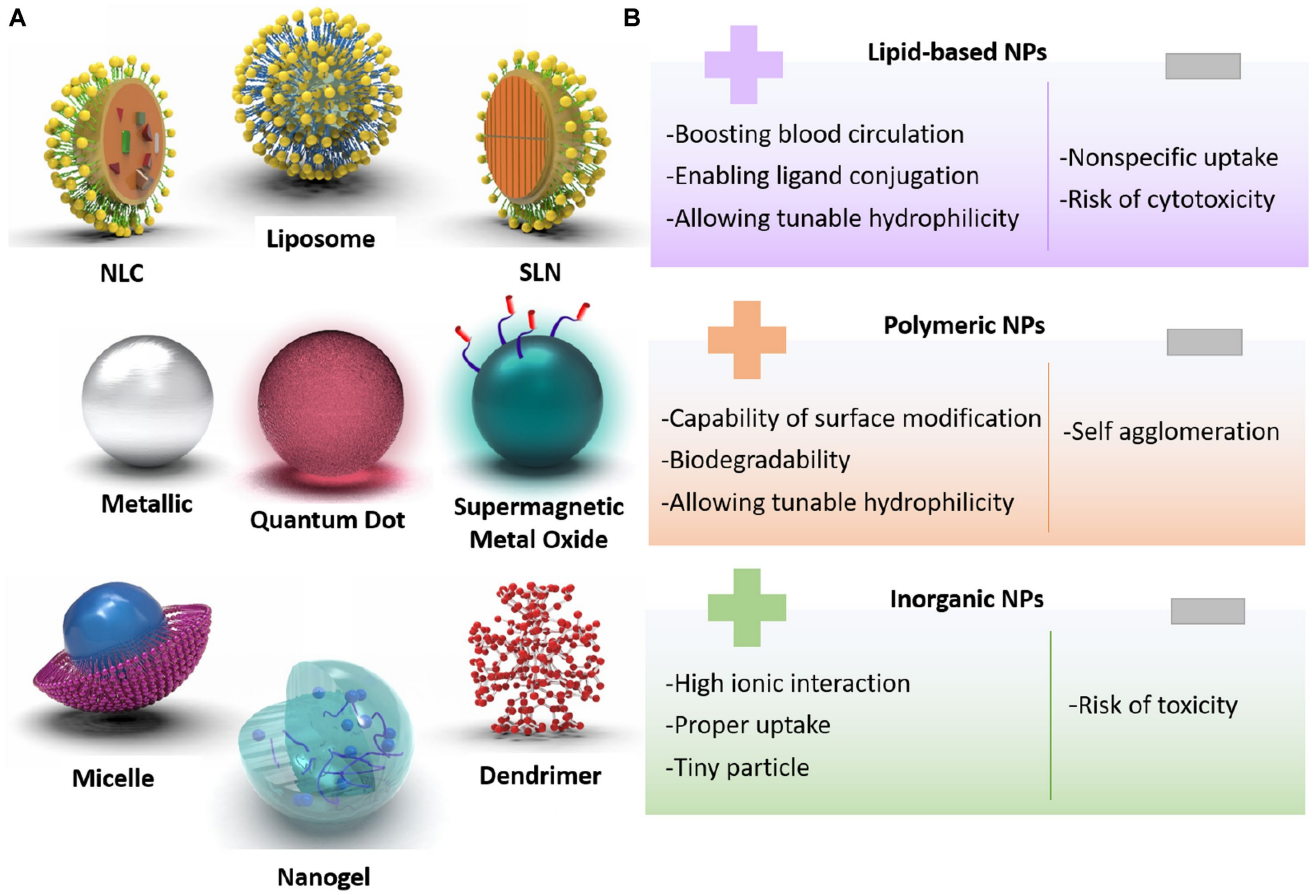
**(A)** Classification of designed nanoparticles toward successful stroke therapy, **(B)** pros and cons of nanoarchitectures for brain-targeted delivery system.

Despite numerous signs of progress in treating ischemic stroke, several challenges are still remained in clinical applications, requiring dominant solutions. First, most of the utilized therapeutics have represented short blood circulation, thereby, they are not capable of crossing the BBB easily. Additionally, stroke therapies need to be improved via deploying novel controllable drug delivery systems. In this era, various nanoarchitectures, including lipid-based compositions (liposomes, lipid-based carriers), polymeric structures (micelles, dendrimers, and nanogels), and inorganic-based configurations (Metallic, super magnetic metal oxides, and quantum dots) could be generated to carry or encapsulate drugs and release them in a targeted region (Tang et al., 2023; Wang et al., 2023; Yu et al., 2023). Figures 3A,B schematically represent different morphologies created using the nanoscience, along with their pros and cons.

One of the prevalent nanovesicles designed for brain therapy is liposomes. The composition of the hydrophilic core with the lipid layer is able to hand over both hydrophobic and hydrophilic drugs in an optimal and qualified release way. Solid lipid nanoparticles (SLNs) and nanostructured lipid carriers (NLC) are prosperous nanocarriers with longer shelf-life and sustained release profiles. Polymeric-based nanoparticle merits are specialty modifications of the surface to produce the carrier with controlled drug release, as well as cell-specific medicine delivery. In the case of brain therapeutic systems, Poly-lactic-co-glycolic acid (PLGA), polylactic acid (PLA), and polyamidoamine (PAMAM) are the most useful polymeric progenitors. Another substance for brain therapy applications is inorganic-based nanostructures, such as iron, gold, and silica. These well-formulated nanoparticles have shown inherent and unique magnetic, optical, electrical, and physical properties that could be utilized in targeted drug delivery and imaging applications (Mitchell et al., 2021; Kasina et al., 2022; Khademolqorani and Banitaba, 2022).

These nanoparticles have shown promising results in preclinical studies, demonstrating improved drug delivery to the brain, and enhanced therapeutic efficacy. As an example, liposomes have been utilized to deliver neuroprotective agents, such as erythropoietin and growth factors to the ischemic brain, thereby reducing infarct size and improving functional recovery. Similarly, polymeric nanoparticles have been used to deliver drugs that target inflammation and oxidative stress, both of which are key components of stroke pathophysiology (Mishra et al., 2018; Matlou and Abrahamse, 2021). The following highlights the advancement of nanodrug delivery systems toward a controllable release of rtPA, antiplatelet, anticoagulants, neuroprotective, anti-inflammatory, and anti-infection agents.

#### 3.2.1. Thrombolytic-based nano medications

The three main useful drugs to retain and restitute blood circulation include thrombolytics, antiplatelets, and anticoagulants. The thrombolytic agents, as clot dissolvers, break down the clots by splitting fibrins and increasing plasminogen content. Whereas, the antiplatelets hinder the platelet agglomeration, and the anticoagulants prevent clot formation.

TPA is the most common generation of thrombolytic drugs that have been promoted to rtPA as a recombinant form of the tPA for clinical use. The effective recombinant tPA is widely used to control thrombosis within 3 h of acute stroke. The thrombolytic drugs have shown different pathways with or without fibrin. Indeed, the presence of fibrin leads to lysing the arginine-valine bond with tPA enzyme, forming the serine protease and finally converting plasminogen to plasmin. Meanwhile, several limitations have been declared for thrombolytic drugs, including systemic side effects, lack of effective targeting, oxidative injury, tPA induced repair fusion, and short half-life (Kleindorfer et al., 2009; Nezu et al., 2010; Fugate and Rabinstein, 2015). With the appearance of nanotechnology, it was expected that new structures would be designed to deal with these limitations. In this regard, the encapsulated thrombolytic agents were introduced to deliver tPA enzymes to target regions at adjustable rate. Using the encapsulation strategy, the tPA activity would be suppressed temporarily in the blood circulation, enhancing its half-life, as well as a longer duration contact with the clot. Correspondingly, better thrombolysis could be achieved with a minimized non-specific bleeding risk (Bruno et al., 2002; Atlantis, 2004; Reeves et al., 2009).

In an effort carried out in this era, Mei et al. (2019), intending to reduce associated oxidative stress after reperfusion, prepared the low-dosage of tPA-loaded in the polyion complex nanoparticles. The encapsulated nanostructure prevented tPA from fast metabolism and eliminated out of the body after long time circulation. The impressive lysis was observed with pristine tPA at both acidic and neutral conditions, while the tPA@nanoparticle displayed no clot lysis activity at neutral pH. It could be referred to complete shielding of tPA after encapsulation into the core of nanoparticles. Meanwhile, the lysis activity of tPA@nanoparticle was significantly increased under the acidic condition at pH 6.2. According to the time course of the tPA activity, the pharmacological efficiency of tPA-loaded nanoparticles remarkably prolonged the half-time of the drug in blood circulation after intravenous administration to mice model. As a result, the mean half-time life of pristine tPA and tPA encapsulated nanoparticles was estimated 8.2 and 72 min, respectively.

**Figure 4:**
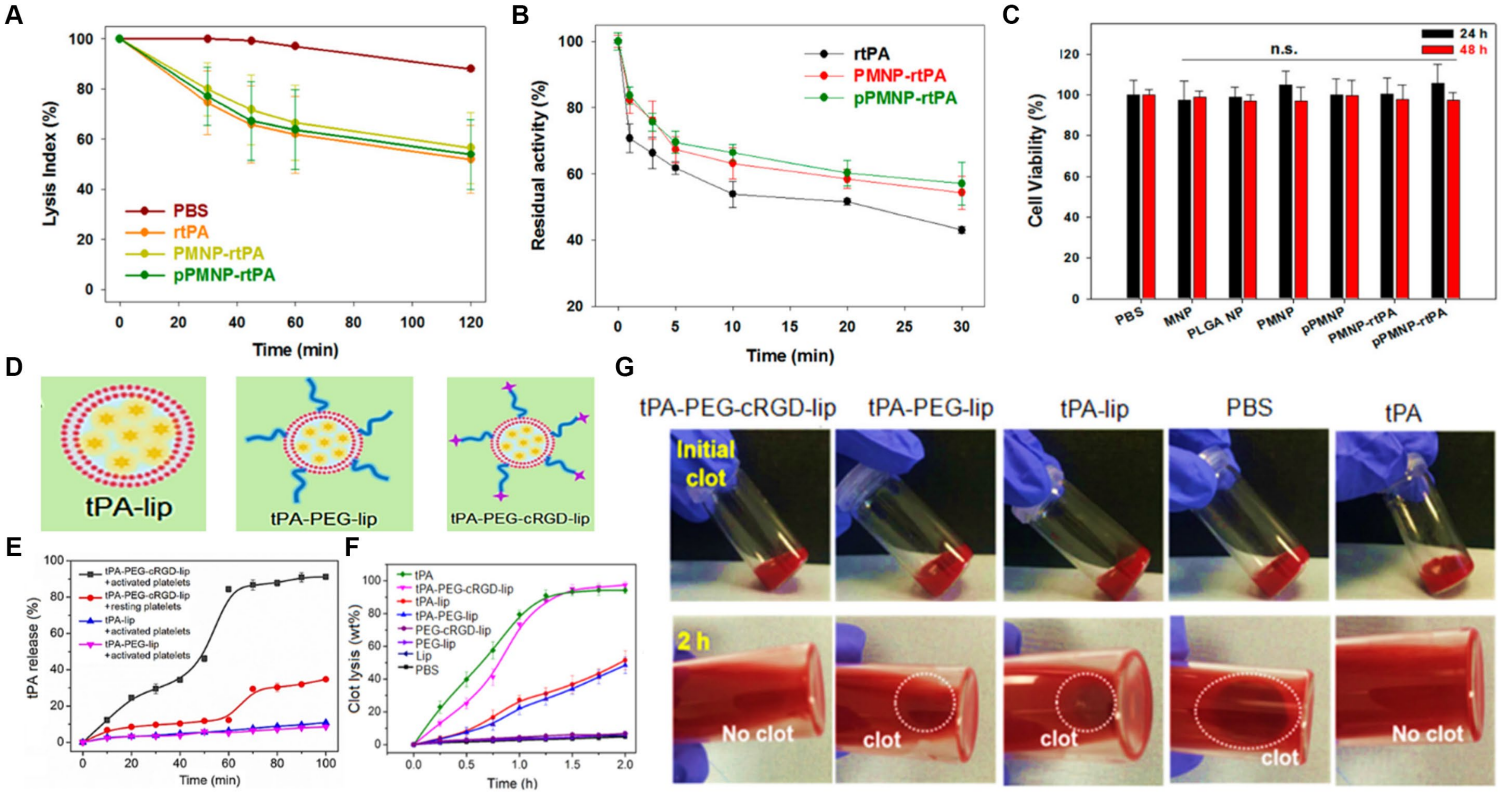
The role of rtPA and peptide-rtPA as a targeted drug strategy; **(A)** lysis index of phosphate buffered saline (PBS), rtPA, PMNP-rtPA, and pPMNP-rtPA at 1.7 μU/mL rtPA dosage, **(B)** the residual amidolytic activity of rtPA agent at 37°C for periods, and **(C)** cell viability of samples according to MTT assay after contacting with NIH 3 T3 fibroblasts cells. Reproduced from Chen et al. (2020) with MDPI Copyright, platelet-sensitive nanocarrier for tPA-targeted release system; **(D)** schematic of tPA-loaded liposomal mechanism, **(E)** tPA release profiles of samples, **(F)** clot lysis after of samples versus time, and **(G)** representative clot images before and after 2 h after incubation with different solutions. Reproduced from Huang et al. (2019) with permission from Elsevier.

Chen et al. (2020) designed magnetic nanoparticles for targeted rtPA delivery system. For this aim, oleic acid-coated iron oxide magnetic nanoparticles (OMNP) were fabricated, then entrapped in poly (lactic-co-glycolic acid). In the next step, the surface of nanoparticles was modified with avidin and peptide/rtPA. Finally, the fibrinolytic activity, residual activity, and cell viability of peptide/rtPA-loaded PLGA magnetic nanoparticles (pPMNP-rtPA) and rtPA-loaded PLGA magnetic nanoparticles (PMNP-rtPA) were investigated (Figures 4A–C). The the lysis index showed no significant difference among all samples at the same drug dosage. It is worth noting that both PMNP-rtPA and pPMNP-rtPA retained fibrinolytic activity for extended blood circulation time. This might regard as the steric hindrance effect of nanocarrier for accessibility of drugs. The biocompatibility of samples was examined through fibroblast cell culturing for 24 and 48 h and applying an MTT assay. As a result, no cell toxicity and meaningful cell viability differences were found for all groups based on ISO No.10993–5. Betala et al. (2020) developed a poly (lactide-co-glycolide)-graft-polyethylenimine (PgP) nanocarrier to heparin delivery in the injured porcine aorta. In agreement with the data, smooth muscle cell proliferation and infiltration were significantly decreased. Based on this, it is predicted that polymeric nanoparticles containing anticoagulants look promising for stroke treatment especially combing with other agents.

To address an effective drug release at the thrombus site, Huang et al. (2019) provided a tPA-loaded liposomes with a diameter of around 165 nm. In this study, PEGylated was applied to boost stability, and the surface of the carrier was modified with conformationally-constrained, cyclic arginine–glycine–aspartic acid (cRGD). The schematic of tPA-loaded liposomal formulations is shown in Figure 4D. The drug release index revealed tPA releasing over 90% by destabilizing of liposomal membrane within 1 h and at *in vitro* conditions (see Figure 4E). As shown in Figure 4F, time-dependent behavior of nanocarrier was investigated after treatment with tPA-PEG-cRGD-lip, tPA-PEG-lip, tPA-lip, PBS, and tPA. Accordingly, the tPA-PEG-cRGD-lip clot lysis activity was lower than tPA at starting timepoint, while the most tPA content of tPA-PEG-cRGD-lip was released after 75 min treatment. Also, the clot dissolution of samples is displayed at Figure 4G, indicating lack of clots in tPA-PEG-cRGD-lip solution after 2 h. In order to have a targeted drug delivery system, compounds sensitive to temperature, ultrasound, and pH can be used to initiate releasing. Accordingly, Liu et al. (2019) prepared magnetoliposomes by integrating the features of magnetic nanoparticles and rtPA drug with thermosensitive liposomes to obtain a rtPA controlled release system at the brain target site under high-temperature conditions.

#### 3.2.2. Neuroprotective nanodrugs

With the aim of supporting the neuronal system against any functional losing and neurodegeneration, neuroprotection is introduced by utilizing various kinds of agents and drugs to inhibit injurious pathophysiological pathways. One of the most common nervous conditions is stroke, requiring a centralized and fast health care treatment. In this field, cytotoxicity, oxidative stress, apoptosis, and even inflammation led to cell death and neuronal injuries. Therefore, hitherto neuroprotection drugs, such as magnesium sulfate, curcumin, glutamate blockers, and melatonin have been introduced. Also, researchers take the merits of nanotechnology for stroke treatment by focusing on blocking proinflammatory cytokines, decreasing lipid peroxidation, lessening immune cell adhesion, obstructing proinflammatory cytokines, and reducing cell apoptosis (Mei et al., 2013).

Osteopontin (OPN), a non-invasive administration, has shown neuroprotective acts through the intranasal rout. Joachim et al. (2014) synthesized the uniform gelatin nanoparticles (GNPs) as a carrier, as well as an OPN peptide as a loaded drug to successfully pass into the brain parenchyma. The presence of gelatin nanosphere provided a OPN targeted delivery condition due to the upregulation of matrix metalloproteinases 2 and 9. Also, the encapsulation technique might be the source of a sustained OPN release from hours to days, along with a protected barrier against protease degradation. In another research, Zhao et al. (2020) proposed a structure to overcome the obstacles and limitations in the reperfusion and treatment of brain hemorrhage injuries. For this purpose, the gallic acid- loaded o-carboxymethyl chitosan nanoparticles (GA-NPs) were prepared through the ionotropic gelation method. In the next step, the *in vitro* and *in vivo* assays were investigated in the oxygen glucose deprivation (OGD). As a result, around half of the drug was released gradually and slowly for 6 h. Cell viability test of GA-NPs has confirmed the significant merits of the loaded GA-NPs in the OGD model. In agreement with pharmacodynamics results, GA-NPs illustrated antioxidant and anti-inflammatory effects on decreasing apoptosis, boosting the neurological deficit, alleviating neural dysfunction, and losing cerebellar infarction size. In this case, the GA-NPs moderated the enhancement of proinflammatory cytokine levels containing TNF-α and IL-β.

Neuroglobin (NGB) is a hemoprotein expressed mainly in the brain especially in the cerebrospinal fluid, central and peripheral nervous system, and retina. This endogenous neuroprotective amino acid supports cell survival against stroke, hypoxic–ischemic brain insults, and neurological disabilities. Blanco et al. (2020) produced hyaluronate nanoparticles through applying both water–oil emulsification and gelation methodologies. The isolated NGB-loaded hyaluronate nanoparticles displayed the spherical shape with an average size less than 150 nm. The hyaluronate nanoparticles successfully passed the BBB after stroke and showed excellent carrier functionality for NGB to the nerve cells. In accordance with *in vitro* release test, NGB protein was released up to 72 h, which could be obtained with encapsulated structure.

**Figure 5:**
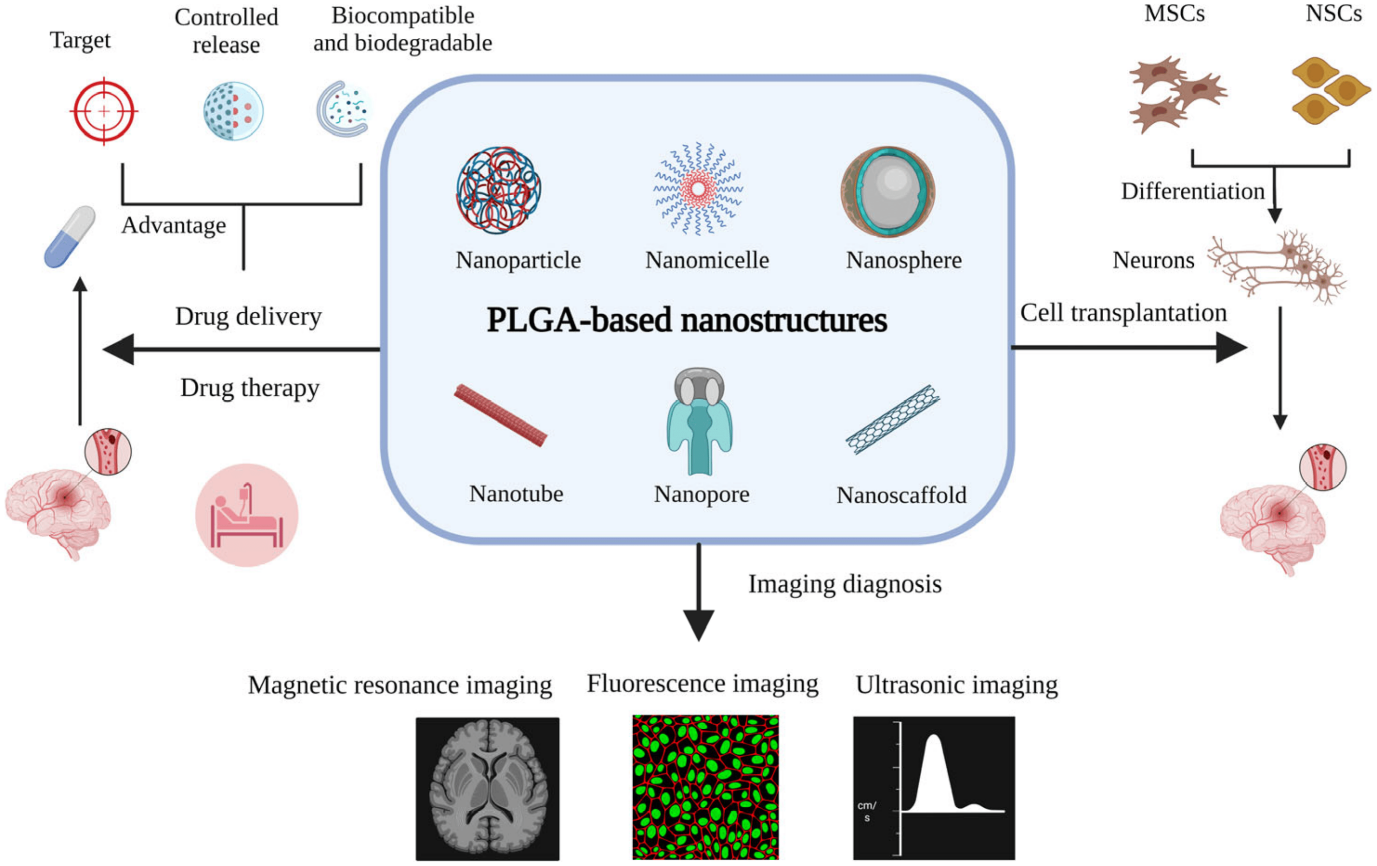
The effective role of PLGA nanostructure in different therapy pathway for ischemic stroke treatment, Reproduced from Yan et al. (2023) with MDPI Copyright.

To provide a successful carrier and overcome the shortcoming of crossing the BBB, PLGA copolymer has been extensively investigated to treat and diagnose the ischemic stroke, as can be seen schematically in Figure 5 and Yan et al. (2023). According to the literature, it can be feasibly synthesized into various nano architectures based on the requirements. PLGA carriers are able to absorb and prevent adverse reactions by crossing the BBB through embedding bioactive agent. Meanwhile, their fantastic characteristics could be effectively enhanced via combining with other materials, such as poly ethylene glycol (PEG). Based on the findings, the addition of PEG to PLGA could increase the water solubility and prolong circulation time. As an example, Li et al. (2019) fabricated wheat germ agglutinin (WGA)-modified PEG/PLGA nanoparticles (WGA-NPs). Transmission electron microscopy (TEM) image of NR2B9c-WGA-NPs illustrated regular spherical shape with an average particle size of around 139 nm. The fluorophore 5-carboxytetramethylrhodamine (5-TAMRA) conjugated to NR2B9c was performed to determine the cellular. delivery against the Calu-3 cell line and neurons. Accordingly, a stronger fluorescence was exhibited for both Calu-3 cells and neurons in the cytoplasm faced with 5-TAMRA-NR2B9c-loaded WGA-NPs. More efficient cellular uptake was observed for 5-TAMRA-NR2B9c-loaded WGA-nanoparticles due to embedding peptide in NPs conjugated with WGA. Also, the neuroprotection activity of NR2B9c-WGA-NPs was investigated after intranasal treatment. The designed nanocarrier released NR2B9c effectively to the brain and remarkably reduced brain infarct volume and neurological deficit level, though NR2B9c-WGA-NPs showed more effective treatment compared with NR2B9c-NPs sample.

PINK1 (PTEN Induced Kinase 1), known as a mitochondrial protein-coding gene, has been involved in neurodegeneration and associated with nervous system disorders like parkinsonism, mitochondrial dysfunction, and stroke. Choi et al. (2023) synthesized PLGA-based nanoparticles incorporated with PINK1 siRNA (PINK1 NPs) to target PINK1. The main purpose of this endeavor was to lessen mitochondrial degradation and mitophagy cell death through quenching PINK1 expression. So, the microglial BV2 cell line was cultured on PINK1 NPs and MTT cytotoxicity assay was performed. As a result, no significant cytotoxicity was identified, and most of NPs were entered the microglia cells. The designed nanoparticles proposed a beneficial neuroprotective and therapeutic agent for stroke treatment due to reducing mitophagy activation and inhibiting microglial activation.

#### 3.2.3. Inhibition of inflammatory after stroke using nanomedications

Cascade of inflammation is commonly triggered after stroke, which could be regulated by various factors, such as cytokines and lymphokines released from damaged brain tissues and reactive oxygen species. Neuroinflammation is one of the main pathological mechanisms originated by cerebral ischemia–reperfusion, mighty causing brain injury. Ischemia also reasons the conversion of microglia (the brain’s resident macrophages) into phagocytosis and the generation of diverse cytotoxic and/or cytoprotective properties. The microglia perform neuroprotection by releasing brain-derived neurotrophic agents, consisting of brain-derived neurotrophic and insulin-like growth factors. Meanwhile, responding to ischemia, the activated microglia generate proinflammatory cytokines, like interleukin-1β (IL-1β), interleukin-6 (IL-6), and oncogenic tumor necrosis factor alpha (TNF-α) (Yeo et al., 2019). While the initial objective of microglia activation is to preserve neuronal cells, its over-activation could result in deleterious inflammation and neuronal death (An et al., 2020). The toll-like receptors (TLRs) are locally expressed in microglia, astrocytes, endothelial cells, and neurons in the brain. TLRs can initiate transcriptional mediators and trigger the expression of proinflammatory factors, neurogenic cytokines and adherence molecules. Moreover, endogenous ligands, such as Hsp70, have been observed to be increased in upregulation after ischemia, with the potential to cause ischemic injury through TLR activation (Qin et al., 2022).

So far, myriad research cases have been dedicated to deploy novel potentiate drugs for surviving and recovering the nerve cells. For example, Purarin, which is a bioactive isoflavone-Cglucoside has shown an effective role as a neuroprotective agent through acting as an antioxidant, along with scavenging the reactive oxygen species. Meanwhile, this agent could not cross the BBB, resulting from its poor water/oil partition coefficient, leading to its low concentration in the brain and so limited therapeutic efficacy. In order to facilitate BBB crossing using this agent, Liu et al. (2021) first prepared the cationic liposomes of puerarin, and neutrophils were employed to carry it across the BBB. The obtained data corroborated the chemotactic behavior of the provided structures in inflammatory endothelial cells. The drug delivery profile was also assessed in various physiological conditions to investigate the function of the proposed structure in inflammatory conditions. PMA and fMLP were applied to simulate different inflammatory environments. Accordingly, in the normal and fMLP conditions, few releases were observed up to 8 h, while a sudden release was discovered for subjecting the structure in PMA for 4 h. The obtained trends could confirm the stable structure in normal homeostatic environment, as well as the blood circulation system, along with the smooth release in the case of reaching the inflammatory condition.

In another research, it was hypothesized that the protective effect of the brain treating drugs could be sustained via using PEGylated lipid nanoparticles. In addition, the effect of binding Fas ligands with the provided nanoparticles on the drug loading efficacy was evaluated. Based on the obtained data, Fas ligands did not show any significant difference compared with the NBP structure on the release profile. Meanwhile, the analysis of cooperative drug targeting was assessed through using the fluorescence imaging *in vivo*, depicting persisting of drug accumulation even 24 h after injection. Notably, the FL-NBP revealed higher accumulation in the ipsilateral side of ischemic brain, proving the design of a promising drug delivery system applicable to brain ischemia (Lu et al., 2014).

#### 3.2.4. Nano-drugs for infection inhibition after stroke

It is widely reported that nanodrugs could improve drug penetration to the location of infection with active microorganisms, while simultaneously limiting systemic toxicity and providing more long-lasting drugs. Hence, the promotion of pharmaceutical efficacy via an improvement in the pharmacokinetic and disease-area specific nervous system of drug bio-distribution and immune-guided elimination of microorganisms could be the best definition in the nanomedicine area associated with infectious conditions. The general aim is to proactively targets and then abrogate sites of ongoing infections, inflammatory conditions, or degeneration (Smith et al., 2018; Anselmo and Mitragotri, 2019; Sun and Gupta, 2020). According to the literature, several nanomedicines have been discussed till now for the therapy, detection, and prophylaxis of communicable conditions (Agreles et al., 2022). The potential for this approach has been gained through enhancing the water sensitivity of a poorly water-soluble drug and subsequent enrichment of drug stabilization to the site of infections. The specific pharmacological targets to endothelium receptors and the utilization of cellular-based nanodrug delivery carriers contribute to facilitating central nervous system (CNS) delivery.

CNS infections, presently in development, include bacteriological meningitis, rabies, malaria, and HIV. There is an active investigation in models and transformational research of human diseases. For instance, a self-assembled quaternary antimicrobial peptide with the chloride conjugate G3R6TAT was reported as a successful strategy in the treatment of *Staphylococcus aureus* and *Cryptococcus neoformans* Meningitis in rabbits (Shuai et al., 2019; Zhen et al., 2019). The particles manufactured crossed the BBB very easily and showed to be as potent as both Vancomycin and Amphotericin B to attenuate the meningeal infection and its sequelae without influencing liver functions or leading to hematological and electrolyte unbalance. These are both used to treat bacterial and fungal conditions with well-known complications. Additional research has demonstrated that the delivery of vancomycin into drug-resistant Staphylococcus pyogenes cultures using folate-bound chitosan Nano-vectors could result in an improvement in medication delivery, highlighting the concept that nanoparticles transport can positively influence the efficacy of therapy for bacterial poly-drug tolerance. This methodology has been of benefit in reducing post-infection oxidative stress in *S. aureus* (Chakraborty et al., 2016).

Vaccination is the optimal therapy for the management of the CNS infection. Nanoparticles could also enhance the immunogenicity and efficacy of vaccines targeted against a few CNS infections. For example, in an attempt performed by Mendoza-Guevara et al. (2021), dendrimer-DNA complexes (dendriplexes) with plasmid immunization constructions derived from rabies viral gluco-protein genes were compounded with a new polyetherimide (PETIM) dendrimer and employed to vaccinate mice that were later contaminated with the classic rabies virus strain. Furthermore, all of the mice that accepted the vaccine with dendriplex recovered from the virtual virus challenges in comparison to 60% of the same mice that accepted the unprepared vaccine. In a further investigation, functioning tri-shell calcium Phosphate (CaP) particles were employed as vehicles for the toll-like receptor 9 ligand CpG and the pathogenic peptide to induction of strong immediately available immune responses as well as prevention of splenomegaly and reduced viral load caused by Friend virus (Mendoza-Guevara et al., 2021). Table 1 summarizes more cases, studying the role of nanomaterials to treat brain infections.

**Table 1:** Recent advancements in synthesis of versatile nanodrugs for treating brain infection.

Drug delivery system	Target	Outcomes	References
BA-PEG-PLGA-based micelles were formulated using rabies virus glycoprotein (RVG29) and Pluronic®P85.	Pneumococcal meningitis	0.5 mg/mL RVG29-PEG-PLGA-PEG-RVG29, 9.5 mg/mL BA-PEG-PLGA-PEG-BA, and 0.01 mg/mL Pluronic^®^P85 could reveal the best formulation for crossing BBB, a satisfactory accumulation, and appropriate therapeutic efficacy. Also, the reported structure depicted low accumulation in the kidney due to the long circulation, as well as a high target performance, proving low toxicity.	Hong et al. (2018)
Lipopolysaccharide (LPS) molecularly imprinted nanoparticles were produced.	*Pseudomonas aeruginosa*	The LPS treated nanoparticles exhibited higher fluorescence intensity for *Pseudomonas aeruginosa*, resulting from the high affinity of LPS and nanoparticles. The LPS binding could also enhance the accumulation in the infected sites, covering the cornea, as well as peripheral tissues.	Long et al. (2016)
Iron-based magnetic nanocarriers were synthesized to transport brain derived neurotropic factor (BDNF) cross BBB.	HIV-I	The proposed structure showed no toxicity. It also could transport about 73% of the BDNF cross BBB, three times higher than free BDNF.	Pilakka-Kanthikeel et al. (2013)
CaFe_2_O_4_@BaTiO_3_ nanoparticles were bounded to siBeckin1 to target the infection of HIV-I.	HIV-I	According to the results obtained, the nano-bound drug could pass the BBB without any changes in cellular viability and eliminate the inflammatory molecules in the infected microglial cells.	Rodriguez et al. (2017)
A liposome-encapsulated glucocorticosteroid, β-methasone hemisuccinate structure was proposed to inhibit Plasmodium falciparum infection.	Cerebral malaria	The performed assays represented the significant reduction of cytokine and chemokine proinflammatory responses, the main result of cerebral malaria infection.	Guo et al. (2014)
Sterically stabilized nanoliposomes loaded with glucocorticosteroids were prepared.	Cerebral malaria	The drug toxicity was reduced for the live cells, using the nanoliposome structure. Additionally, experimental cerebral malaria was effectively prevented even in starting treatment at the late stages of the disease.	Waknine-Grinberg et al. (2013)
Silica-coated gold nanorods were fabricated to suppress brain tumors.	Rabies	The provided nanorods were synthesized considering the shape, size, and surface of glycoprotein, showing an appearance resembling the rabies virus, passing the BBB, and responding to near-infrared laser irradiation to eliminate brain tumors.	Lee et al. (2017)
Paclitaxel-cholesterol complex was prepared and embedded into the RVG15-liposome structure to enhance drug tumor selectivity and performance.	Rabies	According to the performed experiments, proper targeting efficiency, as well as safety for the human brain microvascular endothelial cell line, were observed. The cumulative transport efficiency could also be enhanced from 8.31 to 16.82%, mighty resulting from higher cellular uptake of the structure in the cells.	Xin et al. (2021)
Doxurubicin-conjugated nanoparticles were synthesized to impede the neutrophils’ innate immune response during infection in stroke.	Proinflammatory neutrophils	Neutrophil number, as well as cytokine contents, could be maintained constant up to 72 h after administrating the proposed nanodrug. This selective targeting was able to immune cells for drug delivery and manage inflammatory responses to infection.	Zhang et al. (2019)

Overall, recent advancements regarding nanodrug delivery for treating stroke include the development of smart and multifunctional nanoparticles that can respond to specific stimuli, such as variations in pH or temperature. The pH-sensitive nanoparticles can release drugs in response to the acidic environment of the ischemic brain, resulting in targeted drug delivery and reduced off-target effects. Multifunctional nanoparticles have also been designed to carry multiple drugs or imaging agents, allowing for personalized and precise stroke therapy. According to the promising data, nanodrugs can revolute stroke therapy, but several challenges must be addressed before they can be translated to clinical practice, including further structural optimizations, performing valid clinical trials, evaluating their safety level, and so forth.

## 4. Conclusion and further prospects

In summary, stroke is known as the second-most mortality cause, as well as a major reason for the disability of adults around the world. Therefore, a large amount of expenditure is annually devoted to stroke-based research toward specializing the healthcare teams and progressing more efficient drugs. Accordingly, the healthcare team plays a crucial role in managing stroke cases across pre-hospital, in-hospital, and post-acute discharge stages through providing standard intellectual supportive care. In addition, down-sizing of drugs has led to offering controllable drug delivery systems in the form of liposomes, nanoparticles, lipid nanocarriers, micelles, dendrimers, nanogels, and quantum dots, capable of crossing the blood brain barrier feasibly.

Despite numerous research cases devoted to revealing novel recommendations for standardizing the healthcare service and synthesizing more desirable nanodrug systems, the progress in these eras has yet remained sluggish. Based on the overview provided, education of communities, staff, and patients is recommended to be considered in the first step. Also, the analysis of data attained from patients, as well as implementation of the quality methodology, should be assumed. Accordingly, the protocols are highly suggested to be maintained and updated regularly. To reach the standards, shared decision making and early intervention progress should be focused. In this regard, artificial intelligence is promising to promote decision making and enhance outcomes through generating unprecedented predictive information.

Although nano-therapy can cross the blood–brain barrier and reach the lesion location precisely to achieve therapeutic effects, several challenges must be addressed before they can be translated into clinical practice. First, the design and synthesis of nanodrugs are complicated and require expertise in various fields, including chemistry, materials, and biology. In addition, the nanodrugs’ characteristics, such as size, shape, and surface chemistry, should be optimized to maximize drug loading, delivery, stability, biocompatibility, and targeting efficiency. Third, the safety and toxicity of nanodrugs are critical concerns, as they may have unique properties affect their distribution, metabolism, and toxicity. Therefore, rigorous safety and efficacy testing are key factors, essential to be focused on before the translation of preclinical findings to clinical trials. Moreover, the education required regarding the administration of nanodrugs should be provided for professional healthcare teams. Eventually, the cost of developing and producing nanodrugs is a significant challenge, requiring investment in research and development. Regarding the mentioned challenges, future works on the targeted drug delivery advancement could undoubtedly result in compensating the shortcomings and yielding revolutionary progress to save stroke patients with the least side effects.

## Author contributions

XH: Data curation, Investigation, Validation, Writing – original draft. YQ: Conceptualization, Investigation, Validation, Writing – original draft. CM: Data curation, Investigation, Writing – original draft. FJ: Funding acquisition, Project administration, Supervision, Writing – review & editing. SK: Conceptualization, Investigation, Validation, Writing – original draft. SN: Investigation, Validation, Writing – original draft, Conceptualization.
